# Retinal Diseases Associated with Oxidative Stress and the Effects of a Free Radical Scavenger (Edaravone)

**DOI:** 10.1155/2017/9208489

**Published:** 2017-01-18

**Authors:** Tomomi Masuda, Masamitsu Shimazawa, Hideaki Hara

**Affiliations:** Molecular Pharmacology, Department of Biofunctional Evaluation, Gifu Pharmaceutical University, Gifu, Japan

## Abstract

Oxidative stress plays a pivotal role in developing and accelerating retinal diseases including age-related macular degeneration (AMD), glaucoma, diabetic retinopathy (DR), and retinal vein occlusion (RVO). An excess amount of reactive oxygen species (ROS) can lead to functional and morphological impairments in retinal pigment epithelium (RPE), endothelial cells, and retinal ganglion cells (RGCs). Here we demonstrate that edaravone, a free radical scavenger, decreased apoptotic cell death, oxidative damage to DNA and lipids, and angiogenesis through inhibiting JNK and p38 MAPK pathways in AMD, glaucoma, DR, and RVO animal models. These data suggest that the therapeutic strategy for targeting oxidative stress may be important for the treatment of these ocular diseases, and edaravone may be useful for treating retinal diseases associated with oxidative stress.

## 1. Introduction

Oxidative stress plays a pivotal role in the development and progression of multiple neurodegenerative disorders, including amyotrophic lateral sclerosis (ALS), Parkinson's disease (PD), Alzheimer's disease (AD), and Huntington disease (HD) [1, 2]. Oxidative stress and neurodegeneration are also involved in several eye diseases, for which there are many published reports [3–5]. Aging, gene abnormalities, and excess exposure to exogenous oxidative stressors (e.g., a light exposure) increase oxidative stress in the eye. In this review, we describe the relationship between oxidative stress and retinal diseases, as well as the effects of the free radical scavenger, edaravone.

## 2. Oxidative Stress

### 2.1. Reactive Oxygen Species (ROS)

Oxidative stress is caused by an imbalance between the antioxidant defense system and the production of reactive oxygen species (ROS), including superoxide anion (O_2_^−∙^), hydroxyl radical (^•^OH), hydrogen peroxide (H_2_O_2_), and singlet oxygen (^1^O_2_). In particular, the superoxide anion (O_2_^−∙^) and hydroxyl radical (^•^OH) with an unpaired electron are also known as free radicals. Hydrogen peroxide exhibits a low reactivity, but it can penetrate cell membranes, including the inner and outer membranes of mitochondria. Therefore, hydrogen peroxide (H_2_O_2_) can react with cellular iron and generate hydroxyl radicals, the most reactive form of oxygen, via the Fenton reaction: H_2_O_2_ + Fe^2+^ → ^∙^OH + ^−^OH + Fe^3+^ [6].

These ROS are produced during the processes of several enzymatic and oxidation reactions. The mitochondrial respiratory chain is the main source of ROS production [7]. In the inner membrane of mitochondria, electrons are transported and oxygen is converted into water. Under hypoxic conditions, this process is not performed to completion, resulting in an increased production of superoxide anions (O_2_^−∙^). Nicotinamide adenine dinucleotide phosphate (NADPH) oxidase (NOX) is the source of ROS, derived primarily from superoxide anions (O_2_^−∙^), via enzymatic reactions [8, 9]. As part of the NOX family, seven oxidases (NOX1–5 and Duox1-2) are recognized [10]. Of these, NOX4 can produce both superoxide anions (O_2_^−∙^) as well as hydrogen peroxide (H_2_O_2_) [11, 12]. Nitric oxide (NO) is produced by the sequential oxidation/reduction of L-arginine to L-citrulline by nitric oxide synthase (NOS), which exists in the form of inducible NOS (iNOS), neuronal NOS (nNOS), and endothelial NOS (eNOS) [13]. NO can react with superoxide anions (O_2_^−∙^) and form peroxynitrite (ONOO^−^) which has a highly potent oxidizing and nitrosating ability [14]. This reaction prompts eNOS uncoupling, resulting in an increase in the formation of superoxide anions (O_2_^−∙^) [15]. Moreover, peroxynitrite (ONOO^−^) oxidizes the eNOS cofactor and further promotes eNOS uncoupling [16].

### 2.2. Cigarette Smoking

Cigarette smoke is known as one of the exogenous sources of ROS [17] and contains multiple ROS producers, such as nicotine and cadmium. Nicotine promotes nitric oxide (NO) production and increases proangiogenic factors [18] and cadmium accumulates preferentially in the RPE and choroid and increases ROS production [19]. Moreover, hydroquinone (HQ) is also included in cigarette smoke. HQ is an abundant oxidant in nature, found in processed foods, plastic containers, and atmospheric pollutants. In addition, cigarette smoke extract (CSE) has been shown to induce alterations to mitochondrial integrity, increase in lipid peroxidation, and significant human RPE cell death [20, 21].

Excess light exposure is also included as a source of ROS. The energy contained in a photon of light changes electron orbitals and can break bonds directly.

### 2.3. Light Exposure

Light exposure reduces lipofuscin autofluorescence [22]. Autofluorescence photobleaching is an indication of lipofuscin photooxidation [23]. At a higher level of light exposure, such as after prolonged exposure or being subjected to blue light, RPE disruption occurs in a manner which permanently alters the autofluorescence pattern [24]. Usually, autofluorescence photobleaching recovers after several hours; however, the detailed mechanism remains unclear. Excess light exposure induces cell death in a murine retinal cone cell line (661W) and can cause a disruption in the phagocytotic function of a human retinal pigment epithelial cell line (ARPE-19) [25, 26].

Oxidative stress entails an excess amount of reactive oxygen species (ROS) that leads to oxidative damage to DNA, proteins, lipids, and mitochondria. Mitochondria become progressively more incompetent with age. Therefore, oxidative stress is associated with several age-related diseases. For a detailed summary of the factors affected by ROS, please see the review by Davalli et al. [27].

### 2.4. Endoplasmic Reticulum (ER) Stress

Oxidative stress is closely linked to endoplasmic reticulum (ER) stress [28–31]. During the induction of the unfolded protein response (UPR), ROS are produced by protein disulfide isomerase (PDI), endoplasmic reticulum oxidoreductin (ERO-1), and NADPH oxidase complexes (i.e., NOX4) [32, 33]. ROS are produced during the transfer of electrons from protein thiol to molecular oxygen by ERO-1 and PDI and during protein misfolding due to the depletion of glutathione (GSH) [34]. In addition, after utilizing GSH, thiols interact again with ERO-1/PDI and are reoxidized. These chain reactions then generate further ROS [34]. ROS can also be produced by unfolded proteins independent of disulfide bond formation. Unfolded proteins in the ER can lead to Ca^2+^ release into the cytosol, which then increases ROS formation in mitochondria [35]. ATP depletion caused by protein folding and refolding processes in the ER lumen is also considered to contribute to increased ATP and ROS production by stimulating mitochondrial oxidative phosphorylation.

### 2.5. Inflammation

Oxidative stress is linked to inflammation [36–39]. It has been reported that oxidative stress-induced RPE cell death primarily due to necrosis induces the expression of an inflammatory gene, high mobility group protein B1 (HMGB1) [40]. Moreover, the inflammatory cytokine, tumor necrosis factor- (TNF-) *α*, is also induced in macrophages and healthy RPE cells by the medium of dying cells exposed to oxidative stress [41]. Conversely, proinflammatory cytokines, such as TNF-*α*, interleukin-1*β* (IL-1*β*), or interferon-*γ* (IFN-*γ*), induce intracellular and extracellular ROS production in human RPE cells [42]. Indeed, these proinflammatory cytokines are upregulated in the eyes of patients with glaucoma, age-related macular degeneration, diabetic retinopathy, or retinal vein occlusion [43–46].

In particular, endothelial cells are affected by inflammation. Inflammation induces shifts in the endothelial cell phenotype, increasing the expression of inflammatory mediators, cytokines, and iNOS activation [47]. These events are observed in both RPE cells and endothelial cells. Moreover, RPE interacts with endothelial cells (ECs) directly and can enhance the proangiogenic potential of the ECs, such as proliferation and migration. For example, TNF-*α* upregulates the expression of vascular endothelial growth factor (VEGF), a major angiogenic factor, in RPE cells via the ROS-dependent activation of *β*-catenin [48]. ROS also affects VEGF-stimulated VEGF receptor 2 dimerization and autophosphorylation. Conversely, VEGF further stimulates ROS production through the activation of NOX in endothelial cells [49]. It has been reported that hypoxia-induced microRNA-424 (miR-424), a member of the miR-16 family crucial for the regulation of cell differentiation [50, 51], promotes hypoxia-inducible factor- (HIF-) 1*α* stability in human umbilical vein endothelial cells (HUVECs). This is achieved by inhibiting the expression of a scaffolding protein, Cullin-2, which is essential for the assembly of the HIF E3 ubiquitin ligase complex [52]. ROS also inhibits the activity of prolyl hydroxylase enzymes (PHD) and factor-inhibiting HIF-1*α* (FIH) by reducing Fe^2+^ availability [53]. In addition, endothelial cell apoptosis is triggered by high glucose-induced overexpression of iNOS in RPE cells activating the PKR-like endoplasmic reticulum kinase (PERK) pathway [54].

### 2.6. Nuclear Factor-Erythroid 2-Related Factor 2 (Nrf2)

Nuclear factor-erythroid 2-related factor 2 (Nrf2) is a nuclear transcription factor regulating antioxidant defense. Nrf2 usually exists in the cytosol and interacts with Kelch-like ECH-associated protein 1 (Keap1), an adaptor for a Cullin-3- (Cul3-) based ubiquitin ligase [55]. Under normal condition, the amount of Nrf2 is maintained at lower levels than that of Keap1 and Cul3 proteins. However, under oxidative stress condition, electrophilic agent increases Nrf2 much more than Keap1 and Cul3 proteins, resulting in the accumulation of Nrf2 in the nucleus. In contrast, Keap1 and Cul3 are not changed in their abundance, subcellular localization, and interaction in response to electrophilic stimuli [56]. The increased Nrf2 translocates into the nucleus, dimerizes with Maf proteins, and binds to the antioxidant/electrophile response element (ARE/EpRE) in the promoters of its target genes. These genes encode protective proteins against oxidative stress, including superoxide dismutase (SOD), catalase, glutathione S-transferases (GST), NADPH quinine oxidoreductase (NQO-1), peroxiredoxin (PRX), heme oxygenase-1 (HO-1), and thioredoxin reductase-1 (TXNRD1) [57–59]. Catalase and SOD directly neutralize hydrogen peroxide (H_2_O_2_) and superoxide anion (O_2_^−∙^), respectively [60, 61]. GST and NQO-1 function as a detoxicating enzyme of electrophilic substances and a xenobiotic-metabolizing enzyme, respectively [62, 63]. HO-1 removes toxic heme, producing iron ions (Fe^2+^), carbon monoxide (CO), and biliverdin [64]. Both biliverdin and its reductive form, bilirubin, are potent antioxidants; bilirubin breaks the oxidation chain reaction of polyunsaturated fatty acids [65].

## 3. Edaravone

Oxidative stress is highly complex and is linked to other forms of stress and effects on various cells. There are two strategies for reducing oxidative stress: (1) enhancing antioxidant enzymes and (2) reducing ROS directly.

Edaravone (3-methyl-1-phenyl-2-pyrazolin-5-one, MCI-186, Radicut®) is a free radical scavenger and a drug used to treat acute ischemic stroke [66]. In Japan, edaravone is administered via an intravenous infusion within 24 h of the onset of acute ischemic stroke in patients with lacunae, large-artery atherosclerosis, and cardioembolic stroke.

**Figure 1:**
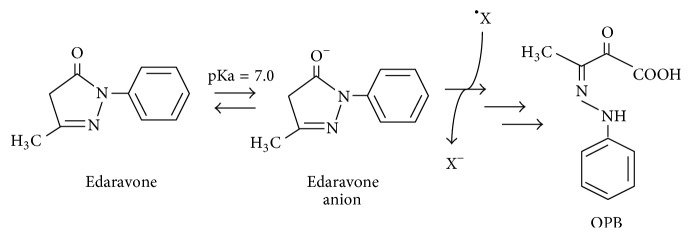
A hypothetical radical-scavenging mechanism of edaravone. Edaravone anion scavenges radicals (^∙^X) to produce anion bodies (X^−^) and edaravone radicals. The final product is 2-oxo-3-(phenylhydrazono)-butanoic acid (OPB), which is without oxidation power.

The hypothetical reaction mechanism of edaravone involves the electron donation to free radicals. The final product derived from edaravone is 2-oxo-3-(phenylhydrazono)-butanoic acid which is without oxidation power (Figure 1)[67–70]. The main metabolites consist of sulfoconjugate and glucuronic acid conjugation. Edaravone quenches hydroxy radicals (^•^OH) and inhibits lipid peroxidation dependent and independent of hydroxy radicals (^•^OH) [67, 71, 72]. Indeed, we demonstrated that edaravone scavenged the intracellular not only hydroxy radicals (^•^OH) but also superoxide anion (O_2_^−∙^) and hydrogen peroxide (H_2_O_2_) [73]. Moreover, edaravone shows a neuroprotective effect against ischemia/reperfusion brain injury and cardiopulmonary resuscitation through a Bax/Bcl-2 dependent antiapoptotic mechanism [74, 75]. Edaravone also ameliorates photoreceptor cell death after experimental retinal detachment through increasing the level of the antiapoptotic Bcl-2 [76].

Edaravone has not only antiapoptotic effect but also anti-inflammatory effect. In the brain with the treatment of middle cerebral artery occlusion, the expression levels of proinflammatory cytokines such as tumor necrosis factor-alpha (TNF-*α*), interleukin-1 beta (IL-1*β*), and inducible nitric oxide synthase (iNOS) were effectively suppressed by edaravone [77]. In addition, the expressions of the inflammatory cytokines TNF-*α* and monocyte chemoattractant protein-1 (MCP-1) in retinal lysates were significantly reduced by edaravone treatment [76].

Edaravone is a low-molecular-weight agent and exerts potency both in water and under lipid soluble conditions [67]. Thus, edaravone is a free radical scavenger with properties of both of vitamins C and E. In addition, edaravone readily crosses the blood-brain barrier, which is unlike other free radical scavengers. These properties of edaravone may be important for its neurovascular protective effects observed in patients with acute ischemic stroke.

Previously, our laboratory demonstrated that combination therapy with normobaric hyperoxia and plus edaravone prevented neuronal damage following focal cerebral ischemia and reperfusion in mice [78]. For a summary of multiple reports on the protective effects of edaravone, please refer to the review by Watanabe et al. [79].

This review describes the relationship between oxidative stress and retinal disease, as well as the effect of edaravone against retinal disease.

## 4. Age-Related Macular Degeneration (AMD)

### 4.1. Pathogenesis and Pharmacological Therapy

Age-related macular degeneration (AMD) is the leading cause of blindness in elderly individuals throughout the world, and approximately 50 million people suffer from AMD worldwide [80]. In addition, the number of patients with AMD continues to increase, and it is estimated that approximately 198 million people currently suffer from AMD [81]. AMD is classified into two types: “dry” and “wet.” In the dry-type AMD, gradual vision loss and drusen, the yellow deposits located under the retina, are diagnostic features [82]. Lipofuscin is the main constituent of drusen and is produced during the reaction of cell metabolites, such as lipid peroxidation [83, 84]. Lipofuscin is deposited when the production of lipofuscin is beyond the disposal capacity of the photoreceptor pigment in RPE [85]. RPE is particularly susceptible to ROS formation due to its high consumption of oxygen, high proportion of polyunsaturated fatty acids, and constant exposure to light. Drusen causes retinal pigment epithelium (RPE) degeneration and “geographic atrophy” appears as feature in eye fundus. When it spreads to the fovea, rapid and severe vision loss occurs. Some dry-type AMD pathology advances to wet-type AMD pathology. The wet-type AMD accounts for 10–15% of AMD patients, and choroidal neovascularization is characterized. The vessels within Bruch's membrane or the sub-RPE space are very weak; therefore, hemorrhage and/or vascular leakage cause damage to the retina leading to further vision loss.

There are several events that occur during the development of AMD, such as oxidative stress, the formation of drusen and RPE dysfunction, apoptosis, activating immune system, senescent loss of homeostatic control, and Bruch's membrane abnormalities. These events are highly complex and involve crosstalk, as well as interaction with each other. As the name indicates, AMD is major ocular disease in elderly individuals [80]. With aging, antioxidant level declines and ROS level increases, ensuring oxidative stress [86]. By aging, macular carotenoids level [87], glutathione S-transferase-1 expression level [88], and vitamin E level [89] are decreased and lipid peroxidation is increased [90]. In contrast, lipofuscin [91, 92], mitochondrial DNA damage in retina [93, 94], advanced lipid peroxidation, and glycation end products [90, 95] are increased. Aging changes the homeostasis of these factors, which means that the rate of AMD development is high in elderly individuals.

Currently, there is no treatment available for the dry-type AMD. In the Age-Related Eye Disease Study (AREDS), antioxidant micronutrients, including *β*-carotene, vitamin C, vitamin E, and zinc, showed a suppressive effect on disease progression [96]. As a therapeutic drug for wet-type AMD, the anti-VEGF antibody is commonly used. Anti-VEGF antibody treatment is the current standard therapy that improves the visual function in patients with wet-type AMD [97]. Patients receive the anti-VEGF antibody treatment via an intravitreal injection at regular intervals. An intravitreal injection is the most common and widely recommended route of drug administration to treat posterior ocular diseases [98]. However, this method is highly invasive and is associated with the risk of infection (0.02 to 1.6%) [99–103]. In addition, the anti-VEGF antibody is very expensive, and the financial burden on patients with the wet type of AMD is extremely high. Therefore, the development of novel therapeutic drug is an urgent need.

### 4.2. The Effects of Edaravone

**Figure 2:**
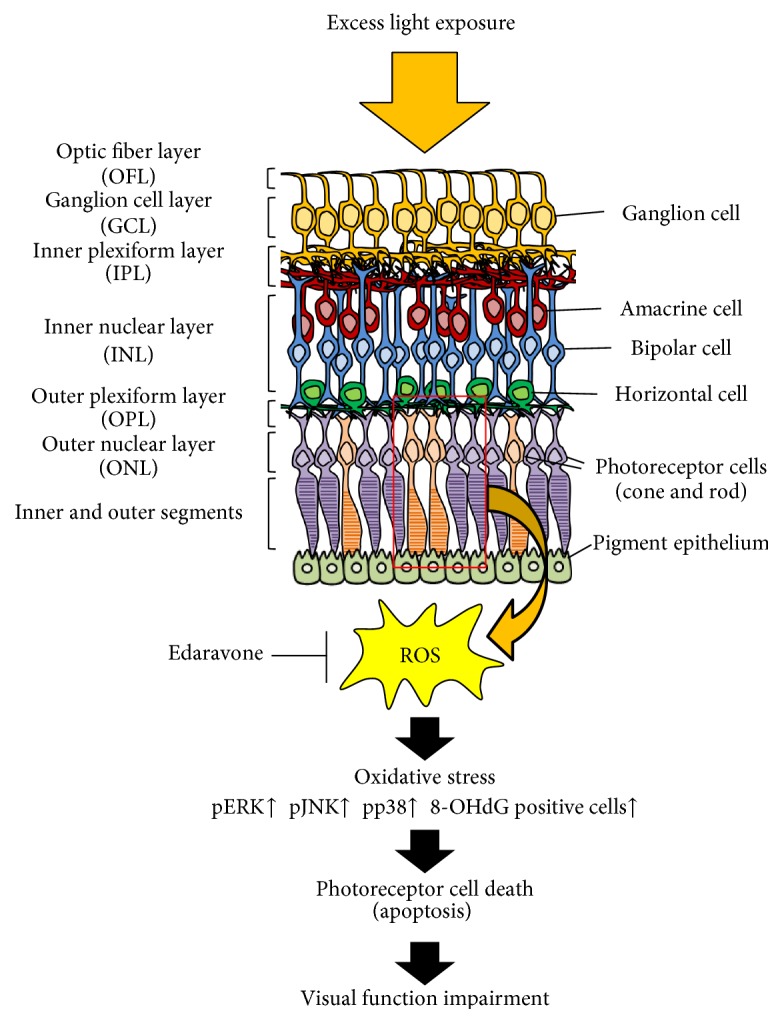
Protective effects of edaravone against light-induced retinal damage. Edaravone scavenges light-induced ROS and rescues light-induced photoreceptor cell death by inhibiting phosphorylated JNK, p38 (but not ERK), and oxidative stress to DNA.

We demonstrated that edaravone is effective against retinal degeneration both in vivo and in vitro [104–106]. A model of light-induced retinal degeneration in mice is commonly used for the evaluation of retinal damage and photoreceptor cell death induced by excess exposure to light [107–109]. Previously, we demonstrated that oxidative stress was involved in light-induced photoreceptor cell death [110–113]. An electroretinogram (ERG) revealed that the intraperitoneal administration of edaravone at a dose of 3 mg/kg (30 min before and just after light exposure) inhibited visual dysfunction five days after light exposure [104]. Moreover, it decreased the number of apoptotic TUNEL-positive cells and was a marker of oxidative damage to DNA, 8-hydroxy-2-deoxyguanosine- (8-OHdG-) positive cells, and the expression of phosphorylated JNK and phosphorylated p38, but not that of phosphorylated ERK, in the whole retina after light exposure [104]. These data suggest that oxidative stress is involved in light-induced retinal degeneration, and the systemic administration of edaravone may slow the progression of photoreceptor degeneration through antioxidative stress [73] and antiapoptotic effects [74–76] (Figure 2). Moreover, this protective effect of edaravone was also observed in N-methyl-N-nitrosourea- (NMU-) induced retinal photoreceptor degeneration in mice, a model of retinitis pigmentosa [114].

Next, we evaluated the effect of the edaravone eye drop, consisting of edaravone-loaded submicron-sized liposomes (ssLips). Eye drop administration is a noninvasive and simple method of the delivery for patients. The protective effects against visual dysfunction and apoptosis induced by light exposure were shown by edaravone-loaded ssLips at a dose that free edaravone could not prevent [105]. Moreover, the edaravone-loaded ssLips used in the study exhibited a low toxicity in ocular cell lines [105].

**Figure 3:**
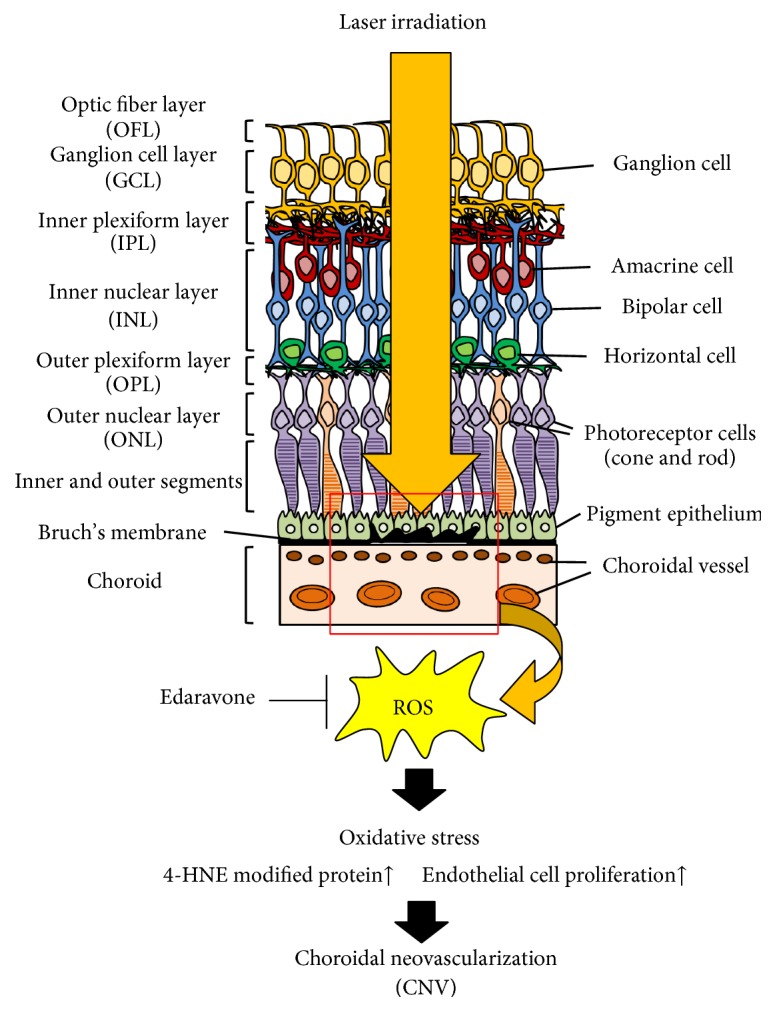
Protective effects of edaravone against laser-induced choroidal neovascularization. Edaravone scavenges laser-induced ROS and rescues laser-induced choroidal neovascularization by inhibiting lipid peroxidation and endothelial cell proliferation.

Edaravone also demonstrated its effectiveness in the wet-type AMD model. A laser-induced choroidal neovascularization (CNV) model was developed as an animal model of wet-type AMD [115]. Laser photocoagulation ruptures Bruch's membrane and induces CNV, which is the main characteristic feature of the disease [116]. Edaravone administered intraperitoneally or intravenously reduced the CNV area and vascular leakage [106]. Surprisingly, edaravone administered intravenously within 24 h after photocoagulation also demonstrated an inhibitory effect [106]. The mechanism of the effect mediated by edaravone is via the reduction of ROS, lipid peroxidation, and VEGF-induced endothelial cell proliferation. Moreover, edaravone was also found to reduce the laser-induced CNV area in the common marmoset, a small monkey [106]. Edaravone demonstrated effectiveness against experimental laser-induced CNV in both rodents, as well as primates, indicating that it may be effective against wet-type AMD characterized by CNV (Figure 3).

Edaravone is already approved for the treatment of acute ischemic stroke. This means that feasibility of clinical application is high because its effectivity and tolerability are very clear. If a combination therapy of anti-VEGF antibody and edaravone exerts a great inhibitory effect against CNV, edaravone would be a powerful candidate for AMD therapeutic medicine and could extend the period of intravitreal injection.

## 5. Glaucoma

### 5.1. Pathogenesis and Pharmacological Therapy

Glaucoma is an optic neuropathy, characterized by retinal ganglion cell (RGC) death, optic nerve head cupping, and visual dysfunction (e.g., scotoma) [117, 118]. Glaucoma is the second most common cause of blindness worldwide [119], and it is expected that over 80 million people will suffer from glaucoma by 2020 [119]. High intraocular pressure (IOP) was considered as a major cause of developing glaucoma; however, in some cases, RGC loss occurred despite a lower IOP [120]. Therefore, IOP reduction alone may be not sufficient for the treatment of glaucoma.

The axons of the RGCs located within the inner retina constitute the retinal nerve fiber layer (RFNL) and merge to form the optic nerve. Therefore, RGC loss causes a loss of RFNL thickness and optic nerve head cupping [118]. The mechanism of RGC loss remains unknown. Similar to age-related macular degeneration, glaucoma is also associated with oxidative stress [121–123]. Previously, our laboratory demonstrated that antioxidant agents including Coenzyme Q10, Astaxanthin, Zeaxanthin, and Docosahexaenoic acid inhibited RGC death induced by H_2_O_2_ or the glutamate analog, N-methyl-D-aspartate (NMDA) [124–127]. In a preclinical study, it was revealed that excitatory amino acids (e.g., glutamate and glycine) were increased and that oxidative stress was one of risk factors for RGC death [128–130]. Moreover, oxidative stress leads to the early impairment of trabecular meshwork (TM) cells which are responsible for aqueous humor outflow and further elevation of the IOP [123, 131]. Indeed, multiple reports have shown that, in the aqueous humor of patients with glaucoma, there were lower levels of antioxidants and elevated markers of oxidative stress [132–134].

In preclinical studies, glutamate antagonists, neurotrophic factors, antioxidants, calcium channel blockers, brimonidine, and nitric oxide synthase inhibitors were shown to exhibit the neuroprotective effects [124, 135–143]. A few agents (e.g., brimonidine and memantine) were evaluated in clinical trials. However, these data have not been conclusive [144, 145].

### 5.2. The Effects of Edaravone

In the model of glaucoma, NMDA-induced retinal damage in mice is commonly used. NMDA induces calcium entry and ROS production, such as NO and superoxide anions (O_2_^−∙^), and results in RGC death [146, 147].

**Figure 4:**
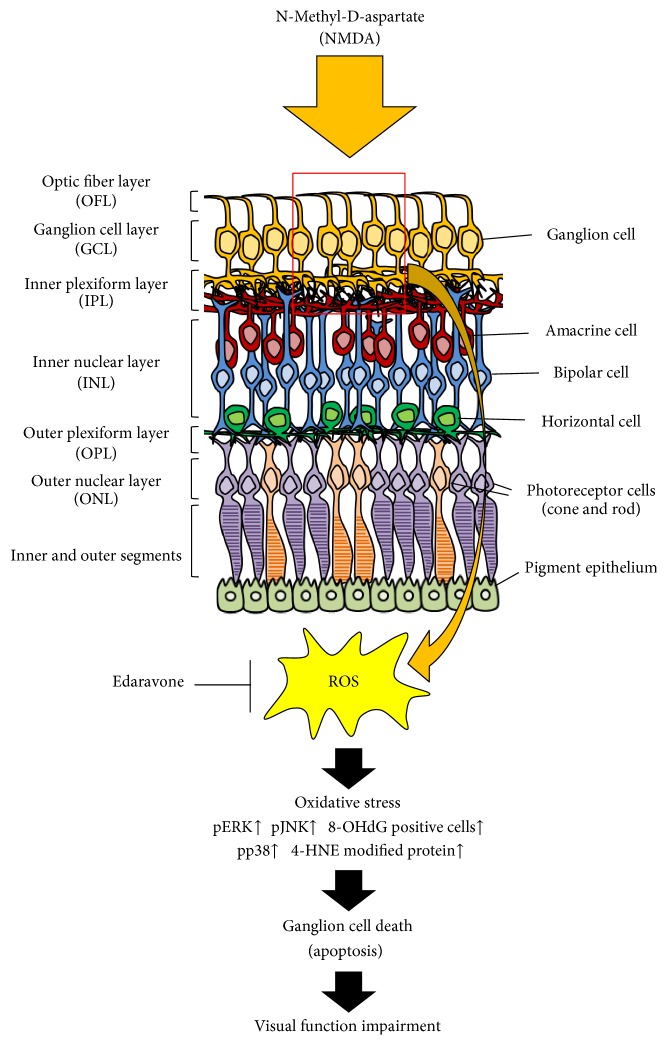
Protective effects of edaravone against NMDA-induced retinal damage. Edaravone scavenges NMDA-induced ROS and rescues NMDA-induced retinal ganglion cell death by inhibiting phosphorylated JNK, p38 (but not ERK), lipid peroxidation, and oxidative stress to DNA.

Edaravone in the form of 5 and 50 nmol intravitreous injections or 1 and 3 mg/kg intravenous injections significantly protected against the NMDA-induced reduction of retinal thickness [73]. Moreover, a 50 nmol intravitreous injection of edaravone decreased the retinal expression of TUNEL-positive cells, markers of oxidative stress (4-HNE and 8-OHdG), and phosphorylated JNK and p38 but not that of phosphorylated ERK (Figure 4) [73]. Another study reported that an intraperitoneal injection of edaravone at a dose of 3 mg/kg also showed potent neuroprotective activity in a hyaluronic acid-induced glaucoma model [148]. Moreover, edaravone-loaded liposomes suppressed the NMDA-induced reduction of retinal thickness [149]. Elevated IOP induces transient ischemic injury. Edaravone also decreased retinal ganglion cell death induced by oxygen-glucose deprivation (OGD) stress in an ischemia-reperfusion injury model in vitro [73].

## 6. Diabetic Retinopathy (DR)

### 6.1. Pathogenesis and Pharmacological Therapy

Oxidative stress is also associated with diabetic retinopathy (DR) [150, 151]. Diabetic retinopathy is one of the most common complications of diabetes mellitus (DM) and the leading cause of blindness and visual dysfunction in working-age populations. Similar to AMD, the number of patients with DM and DR is increasing globally. In the United States alone, 4.1 million people have DR, and the number of patients is expected to double by 2025 [152].

Hyperglycemia induces the excess production of mitochondrial ROS. Increased ROS activates the poly-ADP-ribose polymerase (PARP) pathway and decreases glyceraldehydes 3-phosphate dehydrogenase (GAPDH) activity, which leads to the further activation of the polyol pathway, the protein kinase C (PKC) pathway, advanced glycation end products (AGEs) pathway, and the hexosamine pathway [151, 153, 154]. Under chronic oxidative stress conditions induced by hyperglycemia, Sirt1 and Sirt6 are downregulated and result in endothelial cell senescence [155, 156]. Moreover, increased retinal ROS stabilizes hypoxia-inducible factor-1*α* (HIF-1*α*) and leads to the upregulation of angiogenic genes (e.g., VEGF). As a result, pathological angiogenesis is induced [157–160]. Indeed, the concentration of VEGF was found to be upregulated in the vitreous humor of patients with proliferative diabetic retinopathy, compared to the controls with a macular hole [161]. These pathological vessels can result in a hemorrhage or vascular leakage due to its weakness; therefore, such events cause macular edema, retinal ischemia, and retinal detachment. Furthermore, hyperglycemia accelerates premature endothelial cell apoptosis via mitochondrial dysfunction [162].

Increased hyperglycemia-induced ROS affects both endothelial cells, as well as neuronal cells [163]. Increased ROS also decreases brain-derived neurotrophic factor (BDNF) that regulates axonal growth, synaptic activity, and neuronal survival. The damage to the synaptic transmitter and degradation of neurotrophic factors causes neuronal cell apoptosis and visual impairment [164].

Laser panretinal photocoagulation (PRP) is the primary mode of therapy for neovascularization in proliferative diabetic retinopathy (PDR). PRP treatment was proven to decrease the frequency of severe visual loss in PDR with high-risk characteristics (>50% decrease) [165]. Later, Early Treatment Diabetic Retinopathy Study (ETDRS) demonstrated that the frequency of severe visual loss in severe nonproliferative DR (NPDR) and early PDR was decreased by PRP [166]. However, in mild or moderate NPDR, adverse effects of PRP on visual acuity and visual field were also observed [166]. Therefore, for eyes with macular edema, focal photocoagulation is effective in reducing the risk of moderate visual loss. In recent years, anti-VEGF antibody has received a lot of attention. Ranibizumab (Lucentis®) monotherapy provided better visual outcome than standard focal laser in patients with diabetic macular edema (DME) [167]. Moreover, aflibercept (Eylea®) also provided better visual outcome than standard focal laser in patients with DME [168] and was more effective in improving vision than ranibizumab at worse levels of initial visual acuity [169].

### 6.2. The Effects of Edaravone

The injection of streptozotocin (STZ) is commonly used for the model of type 1 DM. In this model, retinal damage and visual impairment are observed. An intraperitoneal injection of edaravone at a dose of 3 mg/kg was found to significantly attenuate diabetes-induced RGCs death, the upregulation of ROS, ERK1/2 phosphorylation, cleaved caspase-3, and the downregulation of BDNF [170]. These data suggest that oxidative stress is highly involved in diabetic retinal damage and that the systemic administration of edaravone may slow the progression of retinal neuropathy induced by diabetes.

## 7. Branch Retinal Vein Occlusion (BRVO)

### 7.1. Pathogenesis and Pharmacological Therapy

RGC death also occurs under the retinal ischemic conditions during which ROS production is active. Studies have shown that hydroxyl radical (^•^OH) was generated in the retina during ischemic conditions and remained elevated during the reperfusion period [171, 172]. Retinal vein occlusion includes both a branch retinal vein occlusion (BRVO) and central retinal vein occlusion (CRVO). In the United States, it is estimated that approximately 100,000 people suffer from RVO.

Similar to DR, the condition including macular edema, retinal ischemia, and fundus hemorrhage is observed. Retinal ischemia impairs the integrity of the blood retinal barrier, and RVO is a common complication of DR. Blood hyperviscosity is also observed in RVO pathology. In determining blood viscosity, erythrocyte deformability plays a critical role. In RVO patients, ROS production and membrane lipid peroxidation, which are indicative of erythrocyte oxidative stress, are observed and positively correlated with erythrocyte membrane viscosity and deformities [173]. A study in young adult CRVO patients revealed that the serum levels of an antioxidant factor, paraoxonase-1 arylesterase (PON1-ARE) activity, were negatively correlated with hyperhomocysteinemia and lipid peroxidation [174]. Moreover, a glucose-6-phosphate dehydrogenase (G6PD) deficiency was associated with increased erythrocyte vulnerability to oxidative stress and developed CRVO [175]. Clinically, antiphospholipid antibodies have been associated with the development of RVO, since it induces oxidative stress in endothelial cells [176].

Anti-VEGF treatment is applied as the therapy for RVO. An intravitreal injection of triamcinolone acetonide is also applied due to the low cost and longer half-life. However, the effects are not permanent, and there are some risks for the development of adverse events, such as cataract formation and elevated IOP [177].

### 7.2. The Effects of Edaravone

We have reported that the intraperitoneal administration of edaravone at a dose of 1 mg/kg significantly decreased the reduction of retinal thickness and TUNEL-positive cells induced by the ligation of the pterygopalatine artery (PPA) and the external carotid artery (ECA), in a murine retinal ischemic model [178]. Moreover, the intraperitoneal administration of edaravone at a dose of 3 mg/kg lowered a marker of lipid peroxidation, malondialdehyde (MDA), and enhanced superoxide dismutase (SOD) in rodent retinal tissue [179]. MDA is a product of lipid peroxidation and exhibits cytotoxicity. SOD is an antioxidant enzyme that neutralizes superoxide anions (O_2_^−∙^). In addition, edaravone inhibited the retinal ischemia/reperfusion-induced visual dysfunction and apoptosis of retinal neurons within the inner nuclear, ganglion cell, and outer nuclear layers [179]. These data suggest that edaravone scavenges ROS, thereby reducing lipid oxidation, increasing the activity of antioxidant enzyme, and decreasing the extent of cell death and retinal thickness.

In a clinical trial, edaravone following arteriovenous sheathotomy was effective against macular edema associated with a branch retinal vein occlusion (BRVO) and improved the best-corrected visual acuity [180].

## 8. Conclusions

Oxidative stress is highly complex and connected to other factors, such as ER stress and inflammation. Moreover, in retinal diseases, including age-related macular degeneration (AMD), glaucoma, diabetic retinopathy (DR), and retinal vein occlusion (RVO), oxidative stress plays pivotal roles in the development and acceleration of these diseases. In the treatment of these ocular diseases, a therapeutic strategy which targets oxidative stress may be effective.

Edaravone demonstrates protective effects against AMD, glaucoma, DR, and RVO models, suggesting that edaravone may be promising as a novel therapeutic drug candidate.
